# Temporal RT-qPCR-Based Porcine Cardiac Molecular Profiling for Post-Mortem Interval Estimation: Predictive Modeling

**DOI:** 10.3390/ijms27114856

**Published:** 2026-05-28

**Authors:** Vincenzo Cianci, Cristina Mondello, Francisco J. Diaz, Tatyana Zinger, Kristinza Giese, William Ryan, Marketa Češpivová, Daniela Sapienza, Patrizia Gualniera, Alessio Asmundo, Antonino Germanà

**Affiliations:** 1 Department of Biomedical and Dental Sciences and Morphofunctional Imaging, Section of Legal Medicine, University of Messina, Via Consolare Valeria, 1, 98125 Messina, Italy; daniela.sapienza@unime.it (D.S.); patrizia.gualniera@unime.it (P.G.); alessio.asmundo@unime.it (A.A.); 2 Office of the Chief Medical Examiner, Washington, DC 20024, USA; 3 Department of Pathology and Laboratory Medicine, MedStar Georgetown University Hospital, Georgetown University, Washington, DC 20024, USA; william.t.ryan@medstar.net; 4 Department of Anatomopathology, Cliniques Universitaires Saint-Luc, Wolume Saint-Lambert, 1200 Brussels, Belgium; cespivova.marketa@gmail.com; 5 Zebrafish Neuromorphology Lab, Department of Veterinary Sciences, Via Palatucci Snc, University of Messina, 98168 Messina, Italy; agermana@unime.it

**Keywords:** PMI estimation, forensic molecular pathology, mRNA degradation, reference gene stability, postmortem predictive modeling

## Abstract

Estimating the post-mortem interval (PMI) remains a major challenge in forensic pathology, particularly beyond the earliest postmortem phases. RNA-based markers measured by RT-qPCR have been proposed as potential tools for PMI estimation, but their reliability depends on technical robustness, reference gene stability, and data representation. Technical reproducibility was overall satisfactory. However, the candidate reference genes showed different temporal behaviors: ACTB remained relatively stable, whereas RPL4 displayed significant time-dependent drift, resulting in partial instability of the composite reference signal. All target genes were associated with PMI, with HPRT1 and HMOX1 emerging as the most informative markers. Several targets also showed evidence of non-linear temporal dynamics. In predictive analyses, models based on raw Ct values consistently outperformed ΔCt-based models. A parsimonious model based on HMOX1 and HPRT1 showed the most favorable trade-off between interpretability and predictive performance within this exploratory dataset, although prediction error remained non-negligible. These findings suggest that postmortem cardiac transcriptional profiles may contain temporal information useful for PMI-oriented modeling but also show that predictive performance remains limited by reference gene behavior, analytical strategy, and non-negligible estimation error. Nine porcine hearts were stored at 4 °C and sampled at 0, 12, 24, 48, 72, 96, and 120 h. RT-qPCR was performed in technical triplicate for selected target genes (BAX, CASP3, HIF1A, HMOX1) together with additional quantified transcripts (ACTB, RPL4, GAPDH, HPRT1). Technical reproducibility, reference gene stability, temporal trends and predictive performance were assessed using mixed-effects models and predictive models evaluated by leave-one-heart-out cross-validation. Comparative analyses were performed using raw Ct, ΔCt, and ΔΔCt data. Overall, postmortem cardiac RT-qPCR profiling should be regarded as a proof-of-concept framework developed under specific controlled refrigerated conditions. Therefore, further external validation under heterogeneous real-world forensic scenarios and methodological standardization are required before a real-life forensic application.

## 1. Introduction

The estimation of the post-mortem interval (PMI), which is defined as the time interval between death and the discovery or ascertainment of a body, represents one of the most challenging tasks in forensic pathology [1]. Despite its importance, it is well known that, at present, there are only methods of placing PMI within broad time frames depending on circumstances and preservation grade of the corpse, not of determining exact PMI [2].

Although the post-mortem interval is often discussed in relation to different temporal stages, universally accepted boundaries are still not available. From an operational forensic perspective, the early post-mortem (ePMI) period is particularly relevant because PMI estimation in this phase relies on the combined assessment of early post-mortem changes and supravital reactions, including body cooling, livor mortis, rigor mortis, and residual muscle excitability. However, these parameters are influenced by multiple endogenous and exogenous factors, which can reduce their accuracy and reliability. As decomposition progresses, PMI estimation becomes increasingly complex and less amenable to precise reconstruction using early post-mortem signs alone [3,4,5,6].

In particular, intrinsic factors, such as body mass and body surface area, and extrinsic factors, such as environmental temperature, humidity, clothing and insulation, can accelerate or slow postmortem phenomena, reducing the precision of the estimated interval, especially when the time span exceeds 24–48 h [7]. It is no coincidence that since the introduction of these techniques, the adoption of corrective factors and measures such as those related to environmental temperature has become necessary, and a significant margin of error remains [4,5,6,7].

As the elapsed time increases and we move towards more advanced stages, it becomes difficult to identify universally shared standardized tools. In this scenario, forensic entomology represents one of the most consolidated tools: through the analysis of the developmental stages of the entomofauna associated with the corpse and the environment, it allows for the estimation of at least the minimum PMI (mPMI) even in bodies in advanced decomposition [8].

Alongside these, rapid scoring systems based on the observation of decomposition characteristics, such as scoring schemes applied to body regions and integrated with environmental parameters such as accumulated degree days (ADD), have also been proposed. However, their accuracy tends to decrease as the PMI increases and, in some conditions, can lead to overestimations of the elapsed time [9].

In recent years, precisely to overcome the limitations of traditional methods and narrow the estimated time window, research has pushed for more integrated approaches, bringing forensic medicine closer to other disciplines. On the one hand, interest has grown in postmortem microbial succession in different body regions (thanatomicrobiome), with the idea that changes in the composition of microbial environments can reproducibly reflect the stages of degradation [10]. Furthermore, studies are emerging on metabolomics and the assessment of the degradation patterns of various macromolecules (DNA, RNA, and proteins), with initial promising but still heterogeneous results and therefore in need of further confirmation and standardization [11,12,13,14]. In particular, for proteins, it has been hypothesized that specific degradation profiles may depend on both endogenous mechanisms, such as enzymes involved in proteolysis and cell death, and exogenous factors related to putrefaction and entomological activity. However, the variety of tissues, environmental conditions, and protocols makes a conclusive and universally applicable synthesis complex [14].

Within this landscape, RNA has received particular attention as a potential temporal marker, but its interpretation requires caution. Like other biomolecules, RNA undergoes changes after death and is significantly influenced by external and internal variables. Temperature remains a crucial, but not the sole factor: tissue type and individual characteristics, such as BMI, can also significantly alter the speed with which transcript changes or degradation is observed. Furthermore, a significant limitation is the transferability of results obtained in animal models to the human setting, as controlled experimental conditions do not always reproduce the complexity of real-life forensic cases.

From a biological perspective, it is useful to remember that there are different classes of RNA with different roles, which must be distinguished between coding and non-coding RNAs [12]. Among coding RNAs, there is the mRNA. Among the non-coding ones, the most common include miRNA and rRNA. For mRNAs, the literature also describes not only a reduction related to degradation processes but, in some cases, even a relative increase in specific transcripts, especially those linked to cell death pathways. In this context, cellular anoxia resulting from circulatory arrest can activate apoptotic programs (intrinsic and extrinsic), and among the most studied effectors in the experimental setting, Caspase-3 (CASP3), TRP53, and BAX are often cited, although postmortem evidence remains limited compared to what is known in in vivo systems [15].

To transform these observations into useful tools for estimating PMI, however, it is not enough to demonstrate that RNA changes, but it is also necessary to be able to precisely measure these changes within defined time intervals [16].

Reverse transcription quantitative PCR (RT-qPCR) is a sensitive technique used to quantify RNA-derived targets. In this approach, RNA is first reverse-transcribed into complementary DNA (cDNA), which is then amplified and monitored in real time through fluorescence-based detection. The cycle threshold (Ct) reflects the amplification cycle at which the fluorescent signal exceeds a predefined threshold and is inversely related to the initial amount of target cDNA. In gene expression studies, Ct values may be analyzed directly or normalized to reference genes using approaches such as ΔCt or ΔΔCt. However, reliable normalization requires that reference genes remain stable under the specific experimental conditions being investigated [17].

In this sense, RT-qPCR is commonly referred to as the reference method, but it is equally well known that sampling and pre-analytical conditions can alter actual concentrations and influence the final interpretation. This underscores the importance of standardization and, in particular, the use of reference genes for normalization: for a reference to be reliable, it must meet stringent criteria, especially stability of expression under experimental conditions [17,18]. For this reason, as occurred in the present study, new reference genes must be tested, evaluating whether they are suitable for this purpose.

In light of these considerations, the present study aimed to investigate the potential of RT-qPCR-based molecular profiling for PMI estimation by characterizing the temporal behavior of selected transcripts in porcine cardiac tissue under controlled refrigerated conditions. Particular attention was devoted to technical reproducibility, the stability of candidate reference genes, and the comparison of alternative data representations and predictive modeling strategies. Candidate reference genes were also specifically evaluated to determine whether they were sufficiently stable in this experimental setting to support normalization-based analyses.

Importantly, this controlled refrigerated design was intended to provide a standardized methodological setting and should not be considered a direct reproduction of the heterogeneous conditions encountered in routine forensic casework.

## 2. Results

Nine porcine hearts stored at 4 °C and sampled at seven postmortem intervals (0, 12, 24, 48, 72, 96, and 120 h) were analyzed. Three technical replicates of RT-qPCR were performed for each heart-time-gene combination. 

### 2.1. Technical Reproducibility and Reference Gene Stability Across the Postmortem Interval

**Figure 1:**
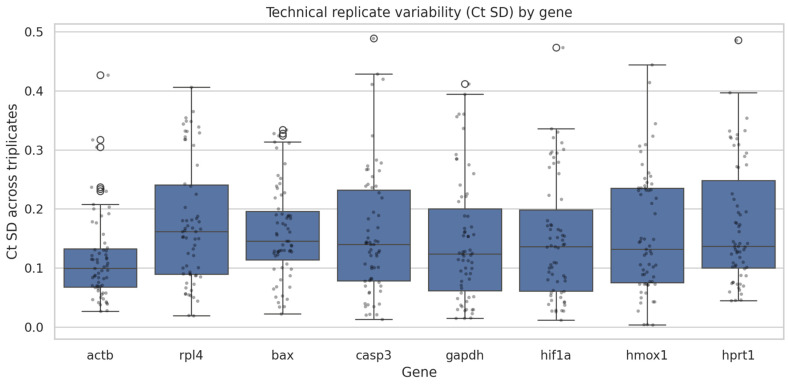
Distribution of the standard deviation of Ct values across technical triplicates for each assay included in the study. Lower values indicate higher technical reproducibility. ACTB showed the lowest replicate dispersion, whereas RPL4 and some target genes displayed a broader distribution of Ct variability while remaining within an overall acceptable technical range.

**Figure 2:**
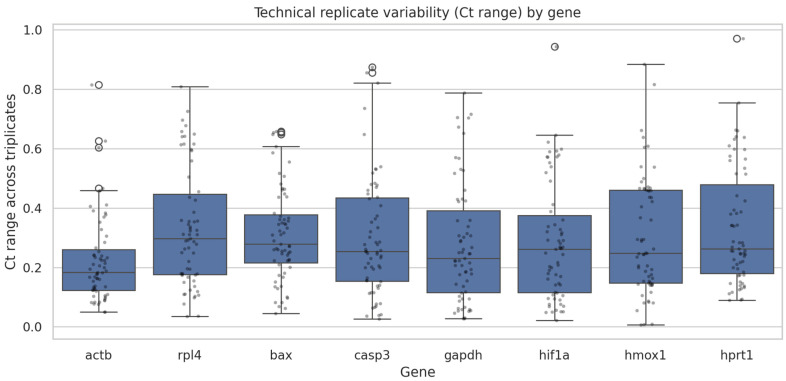
Distribution of the Ct range across technical triplicates for each assay included in the study. The range was used as a complementary measure of within-sample technical dispersion. Consistent with the standard deviation analysis, ACTB showed the narrowest replicate spread, whereas RPL4 exhibited greater variability among triplicates.

The technical reproducibility of the RT-qPCR assays was overall satisfactory across the entire dataset, with no samples classified as technical failures after quality control filtering. Replicate dispersion remained generally low, although some gene-specific differences were observed. In particular, ACTB showed the highest technical consistency, with most samples displaying very limited variability among triplicates, whereas RPL4 exhibited a wider distribution of both Ct standard deviation and Ct range. The remaining target genes showed intermediate behavior, with slightly higher replicate dispersion for some assays, but without evidence of systematic technical instability (Figure 1 and Figure 2, Supplementary Material Table S1).

**Figure 3:**
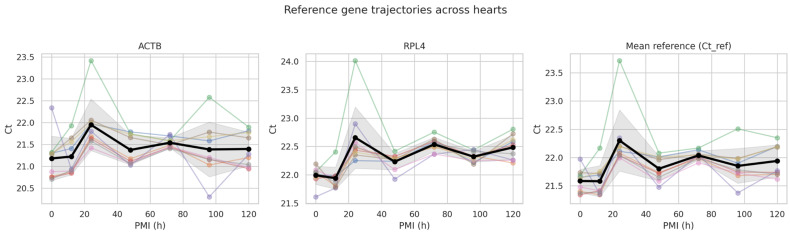
Observed Ct values of ACTB, RPL4, and the composite reference signal (Ct_ref) across the postmortem interval. ACTB remained relatively stable over time, whereas RPL4 showed a progressive increase in Ct values. As a consequence, Ct_ref also displayed a modest temporal drift, indicating that the mean of the two candidate reference genes was not fully invariant across PMI. Each colored line represents one individual porcine heart (H): H1 blue, H2 orange, H3 green, H4 red, H5 purple, H6 brown, H7 pink, H8 gray and H9 yellow. Dots indicate the mean Ct value of each heart at the corresponding postmortem interval, while colored lines connect repeated measurements from the same heart over time. The black line represents the overall mean temporal trend across hearts, and the gray shaded area indicates the confidence band around the mean trend.

When the two candidate reference genes were examined across the postmortem interval, different temporal behaviors emerged. ACTB remained relatively stable throughout the experimental time course, showing only minimal variation and no significant linear association with PMI. By contrast, RPL4 displayed a progressive increase in Ct values over time, indicating a significant temporal drift. As a consequence, the composite reference signal obtained by averaging ACTB and RPL4 (Ct_ref) also showed a tendency to increase with PMI, although this effect was less pronounced than that observed for RPL4 alone (Figure 3, Supplementary Material Table S2A,B).

**Figure 4:**
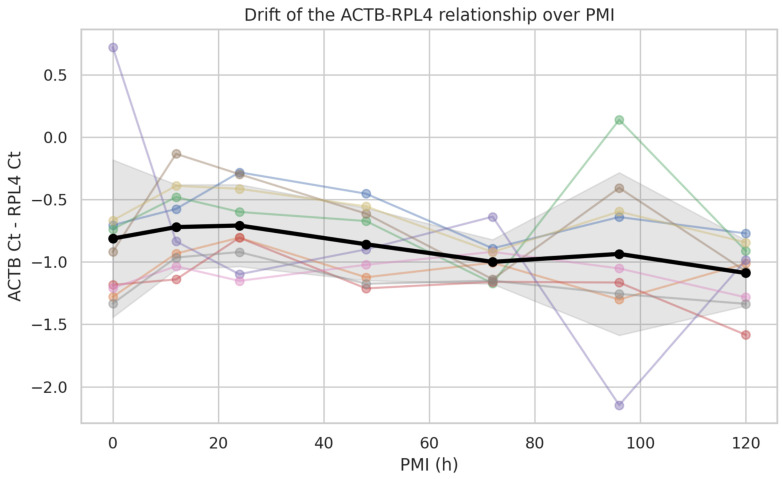
Variation in the difference between ACTB and RPL4 Ct values across the postmortem interval. The non-constant behavior of the ACTB−RPL4 difference indicates a progressive divergence between the two candidate reference genes over time, supporting the conclusion that their reciprocal stability was not maintained throughout the experimental time course. Each colored line represents one porcine heart (H): H1 blue, H2 orange, H3 green, H4 red, H5 purple, H6 brown, H7 pink, H8 gray and H9 yellow. Dots: mean Ct value of each heart at the corresponding postmortem interval. Colored lines connect repeated measurements from the same heart over time. Black line: overall mean temporal trend across hearts. Gray area: confidence band around the mean trend.

This divergence between the two candidate reference genes was further confirmed by the analysis of the ACTB-RPL4 difference, which was not constant over time but instead showed a significant temporal shift, consistent with a loss of stability in their reciprocal relationship during the postmortem interval (Figure 4, Supplementary Material Table S3). 

Overall, these findings indicate that ACTB was the more stable of the two candidate reference genes in this experimental setting, whereas RPL4 appeared more sensitive to postmortem changes. Accordingly, the use of their mean as a normalization factor should be interpreted with caution, since the reference signal itself was not entirely invariant across time (Supplementary Material Table S2A,B).

For this reason, the ACTB–RPL4 mean was retained only as a comparative normalization strategy and not as the primary predictive input. This choice allowed us to preserve the originally defined two-gene normalization framework and to directly evaluate whether a conventional ΔCt approach improved or impaired PMI modeling. The observed temporal drift of RPL4 and Ct_ref was therefore not ignored. Rather, it informed the interpretation of ΔCt-based analyses as secondary and/or sensitivity analyses and supported the prioritization of raw Ct-based models.

### 2.2. Time Course of Target Genes and the Shape of the Relationship with the PMI

**Figure 5:**
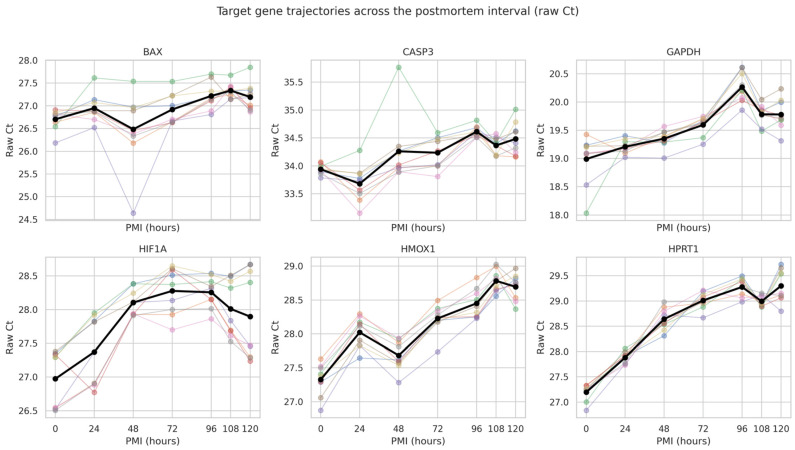
Trajectories of Ct values of the analyzed target genes along the postmortem interval 0–120 h. All transcripts show a progressive increase in Ct values with increasing PMI, although with different intensities between the different genes. HPRT1 and HMOX1 show the most marked temporal gradient, while BAX shows a more contained and dispersed trend. Each colored line represents one porcine heart (H): H1 blue, H2 orange, H3 green, H4 red, H5 purple, H6 brown, H7 pink, H8 gray and H9 yellow. Dots: mean Ct value of each heart at the corresponding postmortem interval. Colored lines connect repeated measurements from the same heart over time. Black line: overall mean temporal trend across hearts.

**Figure 6:**
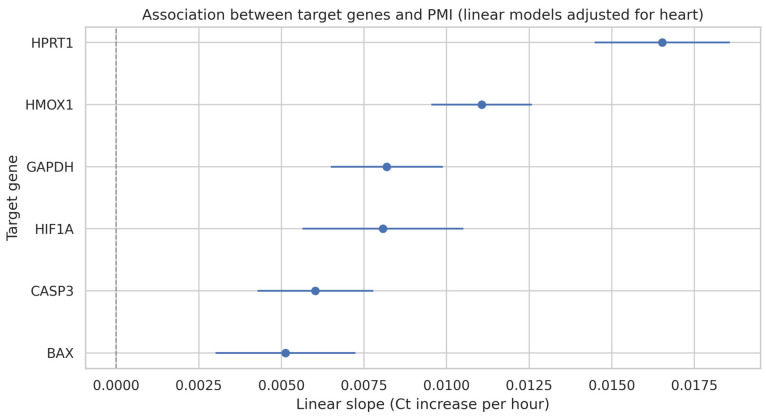
Estimation of the linear association between target gene Ct values and PMI using cardiac-adjusted models. All genes show a positive slope, indicative of an increase in Ct over the postmortem period. The most pronounced effect is observed for HPRT1, followed by HMOX1, while BAX has the smallest linear coefficient.

Target gene analysis conducted on raw Ct values revealed a clear effect of postmortem time for all transcripts examined, although the magnitude and shape of the effect differed across genes. Considering the entire 0–120 h interval, all targets showed a mean increase in Ct values with increasing PMI, consistent with a progressive reduction in detectable signal over time. However, the magnitude of the change was not uniform: HPRT1 and HMOX1 showed the most marked temporal variations, while BAX and CASP3 showed more moderate trends while maintaining a clear association with PMI (Figure 5 and Figure 6, Supplementary Material Table S4).

From a descriptive standpoint, the temporal trajectories of individual hearts showed good overall consistency, despite significant interindividual variability, particularly evident at some intermediate time points. Specifically, HPRT1 exhibited the steepest and most regular temporal gradient, followed by HMOX1, while GAPDH and HIF1A exhibited an intermediate trend. BAX was the gene with the least clear profile, with a relatively wider dispersion and narrower separation between some adjacent timepoints (Figure 5 and Supplementary Material Table S5).

To formally quantify the association with PMI, a linear model with adjustment for the heart was estimated for each gene. All targets showed a positive and statistically significant slope: HPRT1 provided the highest coefficient, followed by HMOX1, while BAX showed the lowest slope of the panel. CASP3, GAPDH, and HIF1A also maintained a significant linear association with PMI, confirming that temporal information is distributed across multiple targets, but with different weighting depending on the gene considered (Figure 6 and Supplementary Material Table S4).

**Figure 7:**
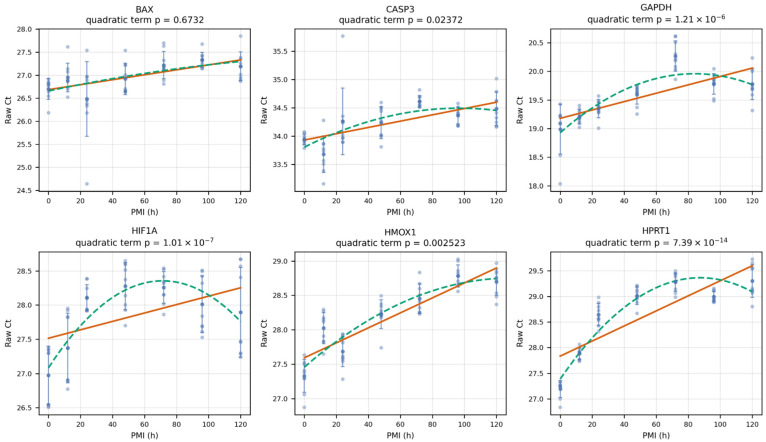
Comparison of the descriptive power of linear and quadratic models applied to the temporal profiles of target genes. For CASP3, GAPDH, HIF1A, HMOX1, and HPRT1, the quadratic model provides a better representation of the temporal dynamics, suggesting the presence of a nonlinear component. For BAX, the improvement over the linear model is limited. Dots represent observed Ct values from individual samples. The solid orange line indicates the linear fit, whereas the dashed green line indicates the quadratic fit. Error bars represent variability around the observed values at each postmortem interval.

Exploring the shape of the relationship between Ct and PMI also showed that, for several targets, a simple linear approximation does not optimally describe the entire temporal profile. Comparison between linear and quadratic models highlighted a significant nonlinear component for CASP3, GAPDH, HIF1A, HMOX1, and especially HPRT1, while for BAX, the quadratic model did not provide a substantial improvement over the linear formulation (Figure 7 and Supplementary Material Table S6). 

Overall, these results indicate that the temporal signal of the targets is not only present but, in several cases, takes on a curvilinear pattern, with an initial phase of more rapid increase and a subsequent tendency to plateau or stabilize in the later phases of the PMI.

Overall, this set of results suggests that the most informative targets, in terms of both amplitude of variation and temporal coherence, are HPRT1 and HMOX1, while GAPDH and HIF1A maintain an intermediate contribution. BAX, although significantly associated with the PMI, appears less robust as an isolated temporal marker. These data therefore support the idea of building subsequent predictive models by privileging parsimonious panels centered on genes with the strongest and most regular temporal signal (Figure 5, Figure 6 and Figure 7, Supplementary Material Tables S4–S6).

### 2.3. Model Predictive Performance and Comparison of Data Representations

**Figure 8:**
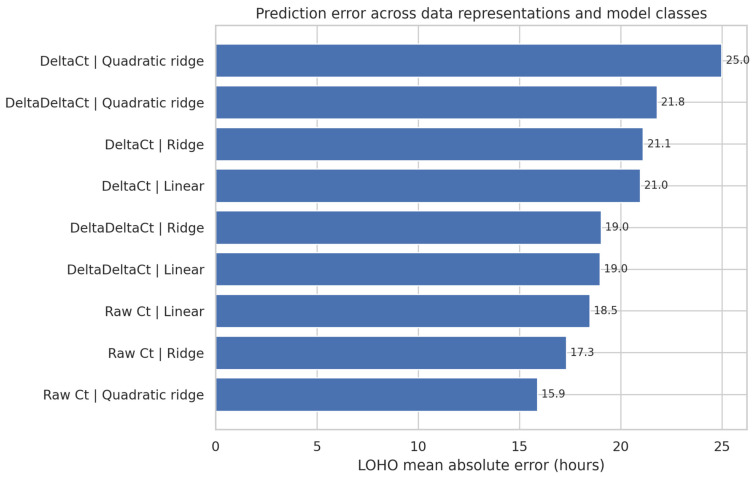
Comparison of predictive performance obtained in leave-one-heart-out validation using different representations of expression data (raw Ct, ΔCt, ΔΔCt) and different model classes. The reported metrics allow a direct comparison of the accuracy and mean error of the different approaches. Overall, models based on raw Ct yield the most favorable performance, while models built on ΔCt are generally less accurate.

The next phase of the analysis was dedicated to evaluating the predictive capacity of the different modeling frameworks for PMI estimation, comparing different representations of the expression data and different classes of models in leave-one-heart-out validation. Overall, the results consistently showed that models built on raw Ct yielded superior performance to those based on ΔCt, while models built on ΔΔCt fell somewhere in between and did not offer a sufficiently clear advantage to justify their use as the primary approach (Figure 8 and Supplementary Material Table S7).

Considering all six targets simultaneously, the best overall result was achieved by the quadratic ridge model applied to the raw Ct, with a mean absolute error of approximately 15.9 h. However, even in this case, calibration was not optimal across the entire time range, and the advantage over simpler models appeared relatively small. The linear and ridge models based on raw Ct still performed better overall than the corresponding approaches built on ΔCt, confirming that, in this dataset, normalization did not improve the information content useful for PMI prediction (Figure 8 and Supplementary Material Table S7).

**Figure 9:**
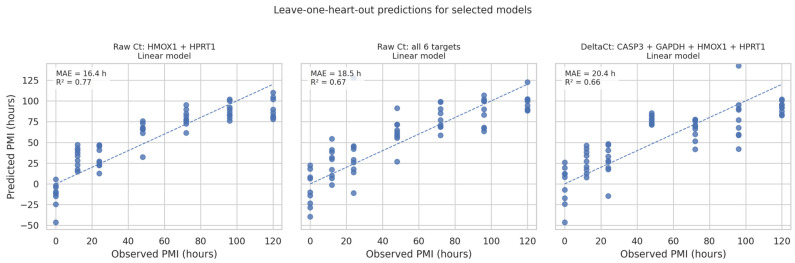
Comparison between observed and predicted PMI values for the models selected as the most representative among those tested. The main panel based on HMOX1 and HPRT1 shows a good ability to follow the general trend of the data, albeit with increasing dispersion at the extremes of the postmortem interval. Models based on larger panels or normalized data show a more marked tendency for predictions to compress towards the central values.

To keep the model as interpretable and tight as possible, a systematic comparison was performed between reduced gene panels. This analysis showed that a simple combination based on HMOX1 and HPRT1 was already capable of achieving competitive predictive performance, with MAE ≈ 16.4 h and R^2^ ≈ 0.77 in leave-one-heart-out validation. Adding additional targets did not result in a substantial improvement and, in some cases, even reduced the robustness of the linear model. In parallel, the best model built on ΔCt required a broader panel, including CASP3, GAPDH, HMOX1, and HPRT1, but still maintained a higher mean error (MAE ≈ 20.4 h) than the parsimonious model based on raw Ct (Figure 9 and Supplementary Material Tables S8 and S9).

Graphical analysis of observed versus predicted PMI values indicated that the primary model based on HMOX1 + HPRT1 captured the overall data trend, but only with moderate precision, showing non-negligible dispersion especially at the extremes of the postmortem interval. The same pattern was more evident in models built with all genes or on normalized data, in which a greater compression of predictions towards the central values and lower accuracy were observed in the earlier or later time windows (Figure 9 and Supplementary Material Table S9).

**Figure 10:**
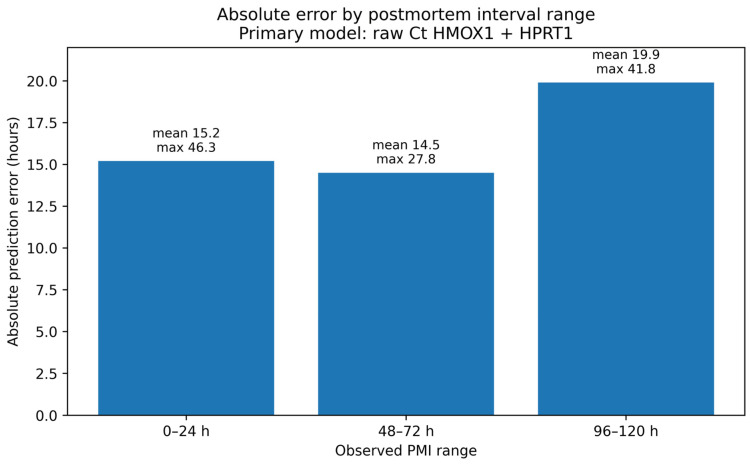
Distribution of the absolute error of the selected primary model across the main PMI time windows. The error is relatively low in the early and intermediate time periods, while it tends to increase in the later PMI samples, indicating lower predictive accuracy in the extreme estimates.

Finally, analysis of absolute error by time window showed that the primary model maintained relatively similar performance in the early and intermediate windows, while the error tended to increase in samples with later PMI. In particular, the mean absolute error was approximately 15.2 h in the 0–24 h window, 14.5 h in the 48–72 h window, and 19.9 h in the 96–120 h window, indicating lower precision in the more extreme estimates of postmortem time (Figure 10).

Overall, these results support further evaluation of a raw Ct-based predictive framework centered on a reduced gene panel. However, the non-negligible MAE and the reduced accuracy at later PMI indicate that the current model should be interpreted as an exploratory proof-of-concept rather than as a definite tool for precise individual PMI estimation.

## 3. Discussion

The present study should be interpreted within a field in which the use of RNA degradation and postmortem transcript variation for PMI estimation is already established. Accordingly, the novelty of this work does not reside in proposing RNA degradation as a new PMI marker and in the simultaneous evaluation of a multi-gene panel for PMI-oriented modeling. Specifically, we evaluated candidate reference-gene stability, compared raw Ct, ΔCt, and ΔΔCt representations, assessed reduced versus broader marker panels, and used leave-one-heart-out validation to estimate internal predictive performance. This approach allowed us to show that normalization based on incompletely stable reference genes may reduce, rather than improve, PMI-oriented model performance in this experimental setting.

The results confirm that the postmortem transcriptional signal contains useful temporal information but also indicate that this information is strongly influenced by the choice of markers, the normalization strategy, and the form of the model used. This interpretation is consistent with the most recent literature, which recognizes the potential of mRNAs in PMI estimation but at the same time emphasizes the heterogeneity of available results and the limited transferability of models across different tissues, species, and experimental conditions [15,19,20].

A first element that clearly emerged concerns the distinction between the technical robustness of the assay and the biological adequacy of the normalization. In our data, the reproducibility of triplicates was overall good, with no evidence of systematic technical instability in the included samples. However, this does not automatically imply that the normalization strategy adopted is optimal. The MIQE guidelines require rigorous validation of reference genes and transparent documentation of analytical conditions, precisely because good technical performance does not in itself guarantee correct biological interpretation of the results [21].

In this sense, the evaluation of reference genes represents one of the most relevant findings of the study. ACTB showed relatively stable behavior along the PMI, while RPL4 showed significant temporal drift. Consequently, the composite Ct_ref signal was also not completely unchanged. This suggests that normalization based on the mean of ACTB and RPL4 does not fully correct for temporal variability and may, at least partially, incorporate it. This finding is consistent with previous studies on postmortem tissues, which have shown that universally stable reference genes do not exist and that their selection must be validated specifically for the tissue, context, and experimental design. In particular, it has been shown that the stability of endogenous controls can differ substantially between heart, brain, and skeletal muscle and that their reliability also varies depending on the postmortem conditions considered [21,22,23].

Accordingly, the continued use of Ct_ref in the present analysis should be understood as a benchmark and sensitivity analysis rather than as evidence that ACTB and RPL4 constitute an optimal reference pair for postmortem cardiac tissue. The inferior performance of ΔCt-based models supports this interpretation and suggests that the composite reference signal may have introduced residual time-related bias rather than improving the predictive information content.

A second notable result concerns the comparison between different representations of expression data. In our models, raw Ct values showed consistently superior predictive performance compared to ΔCt values, while ΔΔCt values did not offer a sufficient advantage to be considered the primary approach. This result does not call into question the theoretical value of normalization; instead, it indicates that in this experimental setting normalization does not improve the available information content, likely because the reference genes used are not fully stable. It is also plausible that, in postmortem samples, raw Ct values also capture global components related to RNA degradation, sample integrity, and extraction yield. This finding is consistent with the literature, which has documented the substantial impact of RNA quality on RT-qPCR measurements in postmortem tissues and the implications of such variability on data interpretation [19].

Regarding biological targets, HPRT1 and HMOX1 were the most informative markers both in terms of variation across the PMI and in terms of their contribution to predictive models. GAPDH and HIF1A showed an intermediate signal, while BAX appeared less robust as an isolated temporal marker. This hierarchy, however, should be interpreted empirically and predictively, not as evidence of a specific postmortem biological program. The available literature suggests that the value of individual transcripts in estimating the PMI strongly depends on the analyzed tissue, the time window, the temperature, and the experimental conditions, and that a universally valid set of transcriptional markers does not yet exist [15,19,20,24].

Our analyses also showed that, for several targets, the relationship between expression and PMI is not optimally described by a simple linear model. For CASP3, GAPDH, HIF1A, HMOX1, and especially HPRT1, quadratic models provided a better fit, suggesting that the dynamics of the transcriptional signal are not constant across the entire postmortem interval. This aspect is also relevant from an applicative perspective, as it indicates that simple linear models may lose information, particularly in later time windows. At the same time, the introduction of moderate model flexibility only partially improved overall performance and did not eliminate the loss of precision at the extremes of the studied range. This finding is also consistent with the idea, well-documented in the literature, that PMI estimation is an intrinsically multifactorial problem, in which biological and pre-analytical variability tends to amplify with increasing postmortem time [15,19,25].

From a predictive perspective, we consider it particularly relevant that a parsimonious model based on two genes, HMOX1 and HPRT1, showed competitive performance compared to larger panels. This suggests that, at least in an exploratory phase, a simpler and more interpretable approach may be preferable to more complex models. However, our data also clearly show that a good correlation does not automatically equate to full forensic utility. The absolute error remains non-negligible, especially at later PMIs, and the model calibration is not uniform across the entire interval considered. For this reason, we believe it is more appropriate to define this work as a proof-of-concept with internal validation rather than as the definitive validation of a model ready for practical application [19,26].

Among the study’s main strengths are the serial sampling of the same hearts across multiple timepoints, the integration of technical quality control, reference gene evaluation, and predictive modeling, and the identification of a small, highly informative panel. These elements allow for a more methodologically complete interpretation than many exploratory PMI studies based on a single level of analysis. The literature shows that reference gene stability and RT-qPCR performance can vary significantly as a function of ante- and postmortem factors. Therefore, the extension of our findings to real-world forensic settings must be considered preliminary [22,25].

Thus, our data indicate that the postmortem cardiac transcriptome can not only provide useful information for PMI estimation but also show that the robustness of this information critically depends on the choice of markers, reference gene stability, and model design. In our experimental setting, raw Ct proved more informative than ΔCt, and a reduced signature based on HMOX1 and HPRT1 provided the best compromise between simplicity, interpretability, and accuracy. Rather than proposing a definitive model, this study defines a useful methodological framework for subsequent studies, which will require the inclusion of independent cohorts, more heterogeneous environmental conditions and additional candidate reference genes before real forensic transferability can be assumed [19,21,22,23,25].

### Risk of Bias and Limitations

The present study has some limitations that should be considered when interpreting the findings. First, the sample size was limited, and although the longitudinal design with repeated sampling increased the amount of temporal information available, the number of biological units remained small. As a consequence, the predictive models should be regarded as internally supported but still potentially sensitive to cohort-specific structure and individual variability.

Second, model assessment was based on internal leave-one-heart-out validation only. This approach is more rigorous than apparent performance estimates and is appropriate for exploratory settings, but it does not replace external validation on an independent dataset. Therefore, the predictive performance reported here should be interpreted as proof-of-concept rather than definitive evidence of generalizability.

Third, in postmortem material, RNA integrity can substantially influence RT-qPCR measurements and may interact with time-dependent changes, potentially affecting both normalized and non-normalized analyses [22,25]. For this reason, part of the temporal signal captured by the models may reflect a combination of transcript-specific behavior and broader RNA quality effects rather than a purely gene-specific biological process.

A further limitation concerns the reference genes used for normalization. Although technical reproducibility was satisfactory, the present analyses showed that ACTB and especially RPL4 were not fully invariant across the postmortem interval, and the composite reference signal displayed residual temporal drift. This means that ΔCt-based analyses should be interpreted cautiously, since normalization may have removed only part of the unwanted variability while simultaneously introducing time-related bias [21,22,23].

Additional sources of bias derive from the experimental setting itself. The study was conducted on porcine cardiac tissue stored under controlled refrigerated conditions, which improved standardization but reduced environmental heterogeneity. While this design is suitable for methodological investigation, it does not reproduce the full complexity of forensic casework, where temperature fluctuations, ante-mortem status, agonal events, tissue-specific pathology, and variable postmortem handling may all influence RNA stability and model performance [19].

Therefore, the findings should not be extrapolated to non-refrigerated bodies, fluctuating environmental temperatures, different tissues, or human forensic cases without external validation in appropriately heterogeneous cohorts.

Finally, under the present conditions, postmortem cardiac RT-qPCR profiles contain exploitable temporal information, but their predictive value depends critically on analytical choices, especially normalization strategy, marker selection and model structure.

## 4. Materials and Methods

### 4.1. Samples and Experimental Design

A total of nine porcine hearts were obtained from pigs slaughtered for human consumption within the commercial food chain. No animals were bred, handled, euthanized, or sacrificed specifically for the purposes of this study. Animals were slaughtered according to routine abattoir procedures by captive bolt stunning, followed by exsanguination. Hearts were collected immediately after death. The first tissue sampling point, corresponding to T0, was performed immediately after heart extraction and before refrigerated transport. After T0 sampling, hearts were placed in clean individual containers and transported to the laboratory under refrigerated conditions at 4 °C within approximately 15 min. During the experimental period, intact hearts were stored under controlled refrigerated conditions at 4 °C. Refrigerator temperature was monitored using a calibrated thermometer. Ambient temperature and humidity at the collection site were not continuously recorded; however, exposure to ambient conditions was limited to the short interval required for heart extraction and immediate T0 sampling. Each heart was subsequently sampled at seven postmortem intervals: 0 (T0), 12 (T1), 24 (T2), 48 (T3), 72 (T4), 96 (T5), and 120 h (T6).

This time window was selected to evaluate whether selected transcripts showed reproducible temporal patterns under a deliberately controlled refrigerated scenario. The design was intended for methodological assessment and was not intended to reproduce the full heterogeneity of forensic casework, where temperature fluctuations, body habitus, agonal factors, pathology, and variable postmortem handling may influence RNA stability and model performance.

At each timepoint, a tissue specimen was collected from the anterior wall of the left ventricle and immediately placed in a 2 mL Eppendorf tube containing 1 mL of RNA later to stabilize RNA and minimize post-collection degradation. Samples were kept at 4 °C for 24 h and subsequently stored at −20 °C until molecular analysis. Each tube was labeled with a unique identifier, including heart ID (H1–H9) and timepoint (T0–T6) to ensure traceability.

All samples underwent RNA extraction, reverse transcription, and RT-qPCR according to the study protocol. For each gene, three technical replicates were run to estimate within-sample technical variability and to obtain a robust mean Ct value for downstream analyses.

### 4.2. Gene Selection Rationale and Reference-Gene Strategy

The gene panel was selected using a literature-informed candidate-gene approach rather than transcriptome-wide screening [19,20,26,27,28,29,30,31,32]. BAX and CASP3 were included as apoptosis- and cell-death-related transcripts previously investigated in postmortem gene-expression studies [32]. HIF1A was included as a hypoxia-related transcript previously explored in forensic postmortem mRNA studies [26,30], whereas HMOX1 was included as a biologically plausible stress-response candidate because of its involvement in oxidative and hypoxia-related stress pathways, rather than as a previously validated PMI marker. GAPDH and HPRT1 were quantified as commonly used transcripts with potential temporal relevance and/or previous application in postmortem gene-expression analyses [20,29,31]. ACTB and RPL4 were selected a priori as candidate reference genes because they represent endogenous control transcripts from different functional classes and have been previously evaluated among candidate reference genes in porcine RT-qPCR studies [31]. However, their stability was not assumed. In accordance with RT-qPCR reporting and normalization principles, candidate reference genes were explicitly evaluated across the postmortem interval before interpreting normalization-dependent analyses [19,29,30,31,32].The composite reference signal, Ct_ref, was calculated as the mean Ct of ACTB and RPL4 only to test the performance of the predefined two-gene normalization strategy. Because RPL4 and Ct_ref showed evidence of temporal drift, ΔCt-based analyses were interpreted as secondary comparative analyses, whereas raw Ct values were retained as the primary representation for PMI-oriented modeling [19,20,26,27,28,29,30,31,32].

### 4.3. RNA Extraction, Reverse Transcription, and RT-qPCR

Total RNA was extracted from cardiac tissue using the Maxwell RSC SimplyRNA Tissue Kit (AS1340, Promega Corporation, Madison, WI, USA), according to the manufacturer’s instructions. RNA concentration was measured spectrophotometrically using a NanoDrop instrument. RNA quality was assessed by electrophoretic visualization on a 1% agarose gel.

Reverse transcription was performed using the High-Capacity cDNA Reverse Transcription Kit (Thermo Fisher Scientific, Waltham, MA, USA). Each reaction was prepared starting from 400 ng of RNA in a final volume of 20 µL. For RT-qPCR analyses, 5 ng of cDNA was used per reaction well.

RT-qPCR reactions were run on an Applied Biosystems 7500 instrument (Thermo Fisher Scientific) using TaqMan Fast Advanced Master Mix (Thermo Fisher Scientific) in a final reaction volume of 10 µL. The following TaqMan assays were used: HPRT1 (Ss03388274_m1), RPL4 (Ss03374067_g1), ACTB (Ss03376563_uH), GAPDH (Ss03374854_g1), HMOX1 (Ss03378516_u1), BAX (Ss03375842_u1), CASP3 (Ss03382792_u1), and HIF1A (Ss03390447_m1). According to manufacturer specifications, these assays operate at an annealing temperature of 60 °C, with expected amplification efficiencies between 90% and 110%.

All reactions were performed in technical triplicate. A no-template control (NTC) was included for each assay and was consistently undetectable.

For each gene and sample, RT-qPCR measurements were summarized as the mean Ct value (Ct_mean) across technical replicates. The final dataset was organized by heart and timepoint and included four primary target transcripts (BAX, CASP3, HIF1A, HMOX1) and four additional quantified transcripts (ACTB, RPL4, GAPDH, HPRT1). Based on the results of the reference gene assessment, ACTB and RPL4 were primarily considered candidate normalization anchors, whereas GAPDH and HPRT1 were evaluated as additional quantified transcripts and, when appropriate, as candidate predictors in multigene models.

### 4.4. Ct Preprocessing, Quality Control, and Data Representation

For each heart, timepoint, and gene, the Ct value used in downstream analyses was calculated as the mean of the available technical replicates. Technical repeatability was summarized at the sample level using the standard deviation (Ct_SD) and range (Ct_range) of replicate Ct values.

The analytical workflow was organized around three complementary data representations. First, raw Ct values were used as the primary representation for reference gene assessment, temporal trend analysis, and predictive modeling. Second, for normalization-based analyses, a composite reference signal (Ct_ref) was calculated for each sample as the mean Ct of ACTB and RPL4, and normalized values were computed as:

ΔCt = Ct(target) − Ct_ref.

Because subsequent reference gene assessment demonstrated incomplete invariance of RPL4 and of the composite Ct_ref signal, ΔCt values were not considered the primary predictive representation. They were retained to evaluate the impact of using the preselected two-gene normalization strategy and to permit direct comparison with raw Ct and ΔΔCt-based models.

Third, for descriptive within-heart comparisons of temporal shape independent of baseline offsets, an additional transformation was calculated as:

ΔΔCt = ΔCt(time) − ΔCt(T0 of the same heart).

In the revised analytical framework of the present study, raw Ct values were considered the primary input for predictive modeling, whereas ΔCt and ΔΔCt were used for comparative and sensitivity analyses.

Reference gene assessment was performed before interpreting normalization-based analyses. Candidate reference genes (ACTB and RPL4) were evaluated using raw Ct values across all hearts and timepoints. In addition to descriptive summaries, stability was examined by analyzing temporal drift of each gene individually, of the composite signal Ct_ref, and of the within-sample difference ACTB − RPL4. These analyses were used to inform the interpretation of normalization-dependent results.

### 4.5. Statistical Analysis

The statistical analysis was conducted in four sequential steps. First, technical reproducibility was assessed by summarizing the dispersion of Ct values across technical triplicates for each gene, using the standard deviation (Ct_SD) and range (Ct_range) of replicate Ct values. Technical replicates were used for quality control and for calculation of mean Ct values but were not treated as biological replicates. The biological experimental unit was the heart (H1–H9).

Second, candidate reference genes were evaluated using raw Ct values. The temporal behavior of ACTB and RPL4, together with the composite reference signal (Ct_ref) and the within-sample difference ACTB − RPL4, was examined across the postmortem interval using linear mixed-effects models, with PMI as a fixed effect and the heart as a random intercept.

Third, the association between target transcripts and PMI was analyzed primarily on raw Ct values, which were considered the main representation in the revised analytical framework. For each target gene, temporal effects were assessed using mixed-effects models with the heart as a random intercept. Both linear and quadratic specifications were examined to evaluate whether transcript trajectories followed a simple monotonic trend or showed evidence of non-linear behavior. ΔCt and ΔΔCt values were also calculated and used as secondary representations for comparative and descriptive analyses.

Finally, predictive models for PMI estimation were developed and evaluated using leave-one-heart-out cross-validation (LOHO), in which all samples from one heart were held out at each iteration and the remaining hearts were used for training. Alternative data representations (raw Ct, ΔCt, and secondarily ΔΔCt) and different model classes were compared, including linear regression, ridge regression, and models incorporating a quadratic representation of predictors when appropriate. Model performance was summarized using mean absolute error (MAE), root mean squared error (RMSE), and coefficient of determination (R^2^). Calibration was additionally evaluated through observed-versus-predicted plots and error distributions across PMI ranges. Reduced multigene panels were also compared to identify parsimonious predictor sets with competitive performance.

All statistical analyses were performed in Python 3.13.5, using pandas 2.2.3, numpy 2.3.5, scipy 1.17.0, statsmodels 0.14.6, scikit-learn 1.8.0, matplotlib 3.10.8, and openpyxl 3.1.5. Artificial intelligence-assisted tools were additionally used to support code refinement and figure preparation. All outputs were critically reviewed, verified, and revised by the authors before inclusion in the manuscript.

## 5. Conclusions

This study suggests that postmortem cardiac RT-qPCR profiles can provide informative temporal signals for PMI-oriented modeling under controlled refrigerated conditions. Among the transcripts examined, HMOX1 and HPRT1 emerged as the most informative markers, and a parsimonious model based on these two genes provided the most favorable exploratory trade-off between interpretability and predictive performance, although with non-negligible estimation error.

The present findings also indicate that model performance is strongly influenced by the analytical strategy adopted. In this dataset, raw Ct-based models consistently outperformed ΔCt-based approaches, whereas the candidate reference genes were not fully stable across the postmortem interval, limiting the robustness of normalization-based analyses.

These results support a proof-of-concept role for postmortem cardiac RT-qPCR in PMI estimation, but they do not yet support the use of this framework as a validated forensic tool.

Further studies should include independent validation cohorts, broader and more realistic environmental conditions, human forensic samples where appropriate, and additional candidate reference genes to improve robustness and assess potential applicability under real-life forensic conditions.

## Data Availability

The original contributions presented in this study are included in the article. Further inquiries can be directed to the corresponding authors.

## Supplementary Materials

The following supporting information can be downloaded at: https://www.mdpi.com/article/10.3390/ijms27114856/s1.
