# Supplementary material for: Temporal RT-qPCR-Based Porcine Cardiac Molecular Profiling for Post-Mortem Interval Estimation: Predictive Modeling
Source: Int J Mol Sci. 2026 May 28;27(11):4856. doi: 10.3390/ijms27114856 (PMC13256687; doi:10.3390/ijms27114856)
Supplement: Supplementary file 1 [file ijms-27-04856-s001.zip › ijms-4320629-supplementary.pdf]

SM1

Gene	N	CtSD_mean	CtSD_median	CtSD_IQR	CtSD_p95	CtRange_mean	CtRange_median	CtRange_IQR	CtRange_p95	Pct_SD_le_0_25	Pct_Range_le_0_5
ACTB	63	0.117	0.100	0.065	0.237	0.224	0.183	0.137	0.466	95.200	95.200
BAX	63	0.177	0.158	0.141	0.413	0.340	0.267	0.259	0.775	84.100	73.000
CASP3	63	0.190	0.162	0.134	0.455	0.361	0.288	0.250	0.800	82.500	68.300
GAPDH	63	0.162	0.143	0.110	0.372	0.309	0.265	0.201	0.708	88.900	79.400
HIF1A	63	0.213	0.180	0.187	0.480	0.402	0.312	0.332	0.886	73.000	60.300
HMOX1	63	0.180	0.167	0.126	0.381	0.342	0.289	0.246	0.721	82.500	71.400
HPRT1	63	0.196	0.182	0.119	0.412	0.378	0.314	0.232	0.796	77.800	65.100
RPL4	63	0.188	0.165	0.113	0.444	0.353	0.287	0.220	0.801	82.500	76.200

Supplementary Material SM1: Summary statistics of technical reproducibility across RT-qPCR assays. Descriptive statistics of Ct standard deviation and Ct range across technical triplicates for each assay, including mean, median, interquartile range, upper percentiles, and the proportion of samples meeting predefined thresholds of acceptable technical reproducibility.

SM2A

Variable	N	Mean	Median	SD	CV_percent	Min	Max
ACTB	63	21.434	21.433	0.494	2.300	20.302	23.417
RPL4	63	22.350	22.346	0.349	1.600	21.497	23.194
Ct_ref	63	21.892	21.884	0.332	1.500	21.164	22.665
ACTB-RPL4	63	-0.916	-0.942	0.536	-58.500	-2.494	0.729

Supplementary Material SM2A: Descriptive statistics of candidate reference genes and composite reference signal. Summary statistics for ACTB, RPL4, Ct_ref, and ACTB−RPL4, including mean, median, standard deviation, minimum, and maximum values across the full dataset.

SM2B

Variable	Intercept	Slope_per_h	CI_low	CI_high	p_value
ACTB	0	0.000	-0.002	0.003	0.720
RPL4	0	0.003	0.001	0.005	0.001
Ct_ref	0	0.002	-0.000	0.004	0.065
ACTB-RPL4	0	-0.003	-0.005	-0.001	0.011

Supplementary Material SM2B: Linear association between candidate reference genes and postmortem interval. Results of linear models testing the association between ACTB, RPL4, Ct_ref, and ACTB−RPL4 with the postmortem interval, including estimated slope, confidence interval, and p-value for each variable.

SM3

PMI_h	N	ACTB_mean	ACTB_sd	RPL4_mean	RPL4_sd	Ct_ref_mean	Ct_ref_sd	Diff_mean	Diff_sd
0	9	21.179	0.508	21.991	0.160	21.585	0.206	-0.812	0.631
12	9	21.459	0.406	22.076	0.253	21.768	0.260	-0.617	0.542
24	9	21.398	0.538	22.245	0.318	21.821	0.372	-0.847	0.546
48	9	21.474	0.575	22.326	0.274	21.900	0.305	-0.852	0.589
72	9	21.395	0.425	22.416	0.417	21.905	0.213	-1.021	0.567
96	9	21.603	0.571	22.482	0.423	22.042	0.322	-0.879	0.785
120	9	21.530	0.476	22.911	0.218	22.220	0.243	-1.381	0.575

Supplementary Material SM3: Time-point summary of candidate reference genes across the postmortem interval. Mean and dispersion measures of ACTB, RPL4, Ct_ref, and ACTB−RPL4 at each postmortem time point, providing a detailed overview of their temporal behaviour across the experimental series.

SM4

Gene	Mean_Ct	Median_Ct	SD_Ct	Min_Ct	Max_Ct	Linear_slope_Ct_per_h	Slope_CI_low	Slope_CI_high	p_linear	Adj_R2_linear	Adj_R2_quadratic	p_quadratic	Delta AIC_linear_minus_quadratic
BAX	26.972	26.962	0.476	24.641	27.847	0.005	0.003	0.007	0.000	0.466	0.489	0.068	2.062
CASP3	34.226	34.234	0.418	33.155	35.766	0.006	0.004	0.008	0.000	0.527	0.519	0.706	-1.826
GAPDH	19.567	19.569	0.463	18.034	20.615	0.008	0.006	0.010	0.000	0.637	0.654	0.061	2.286
HIF1A	27.842	27.972	0.830	26.138	28.964	0.021	0.017	0.025	0.000	0.602	0.827	0.000	58.858
HMOX1	28.170	28.179	0.526	26.226	29.024	0.009	0.007	0.011	0.000	0.621	0.617	0.743	-1.868
HPRT1	26.571	26.622	0.723	24.968	28.197	0.017	0.013	0.020	0.000	0.562	0.650	0.001	18.673

Supplementary Material SM4: Association of target genes with PMI: descriptive statistics and linear model parameters. Summary table of target genes with descriptive statistics of Ct values and results of linear models versus PMI, including estimated coefficient, confidence interval, and p-value.

SM5

Gene	PMI_h	mean	median	std	min	max
BAX	0	26.703	26.789	0.225	26.183	26.912
BAX	24	26.950	26.882	0.308	26.522	27.614
BAX	48	26.484	26.457	0.811	24.641	27.537
BAX	72	27.258	27.331	0.242	26.962	27.584
BAX	96	27.131	27.084	0.194	26.872	27.504
BAX	120	27.426	27.505	0.200	27.081	27.664
BAX	12	26.842	26.876	0.223	26.356	27.076
CASP3	0	33.858	33.903	0.231	33.426	34.168
CASP3	24	34.036	33.957	0.267	33.728	34.541
CASP3	48	34.224	34.152	0.237	33.927	34.701
CASP3	72	34.371	34.344	0.237	34.059	34.846
CASP3	96	34.594	34.561	0.270	34.195	35.153
CASP3	120	34.800	34.717	0.536	34.069	35.766
CASP3	12	33.697	33.727	0.396	33.155	34.449
GAPDH	0	19.194	19.162	0.410	18.606	19.975
GAPDH	24	19.334	19.356	0.432	18.873	20.030
GAPDH	48	19.424	19.427	0.499	18.695	20.164
GAPDH	72	19.691	19.826	0.439	18.893	20.102
GAPDH	96	19.837	19.859	0.352	19.381	20.336
GAPDH	120	20.312	20.305	0.199	20.094	20.615
GAPDH	12	19.178	19.160	0.437	18.034	19.619
HIF1A	0	26.668	26.728	0.448	26.138	27.600
HIF1A	24	27.366	27.355	0.275	26.831	27.731
HIF1A	48	28.016	28.085	0.159	27.775	28.273
HIF1A	72	28.286	28.257	0.192	28.023	28.581
HIF1A	96	28.503	28.513	0.237	27.943	28.782
HIF1A	120	28.739	28.758	0.168	28.339	28.964
HIF1A	12	27.313	27.298	0.291	26.920	27.918
HMOX1	0	27.562	27.613	0.599	26.226	28.067
HMOX1	24	27.921	27.946	0.277	27.595	28.353
HMOX1	48	27.979	28.023	0.359	27.372	28.434
HMOX1	72	28.231	28.212	0.418	27.488	28.781
HMOX1	96	28.684	28.653	0.267	28.382	29.024
HMOX1	120	28.797	28.825	0.124	28.611	28.949
HMOX1	12	27.753	27.834	0.451	26.784	28.290
HPRT1	0	25.422	25.439	0.359	24.968	26.169
HPRT1	24	26.198	26.256	0.421	25.632	26.777
HPRT1	48	26.907	26.873	0.287	26.554	27.449
HPRT1	72	26.995	26.954	0.252	26.636	27.388
HPRT1	96	27.029	27.066	0.349	26.592	27.549

Gene	PMI_h	mean	median	std	min	max
HPRT1	120	27.289	27.218	0.613	26.433	28.197
HPRT1	12	26.157	26.160	0.251	25.716	26.536

Supplementary Material SM5: Time-point summary of target genes across the postmortem interval. Descriptive statistics of Ct values for each target gene at each experimental time point, providing a detailed view of temporal progression and inter-individual variability.

SM6

Gene	AIC_linear	AIC_quadratic	BIC_linear	BIC_quadratic	Adj_R2_linear	Adj_R2_quadratic	Quadratic_term_coef	Quadratic_term_p	ANOVA_p_model_comparison
BAX	54.799	52.737	76.230	76.311	0.466	0.489	0.000	0.068	0.068
CASP3	30.924	32.750	52.355	56.325	0.527	0.519	-0.000	0.706	0.706
GAPDH	26.940	24.654	48.372	48.228	0.637	0.654	-0.000	0.061	0.061
HIF1A	95.533	36.676	116.964	60.251	0.602	0.827	-0.000	0.000	0.000
HMOX1	46.652	48.520	68.083	72.094	0.621	0.617	-0.000	0.743	0.743
HPRT1	81.020	62.347	102.451	85.922	0.562	0.650	-0.000	0.001	0.001

Supplementary Material SM6: Comparison between linear and quadratic models for target genes. Results of the comparison between linear and quadratic models fitted to individual target genes, including fit indices and tests of model improvement to assess non-linear temporal behaviour.

SM7

Representation	Model	MAE_h	RMSE_h	R2	Bias_h	Calibration_slope	Calibration_intercept
Raw Ct	Quadratic ridge	15.886	19.938	0.769	0.837	0.815	10.686
Raw Ct	Ridge	17.317	22.899	0.696	1.411	0.757	14.307
Raw Ct	Linear	18.477	23.961	0.667	1.634	0.808	11.849
DeltaDeltaCt	Linear	18.969	22.993	0.693	1.385	0.795	12.726
DeltaDeltaCt	Ridge	19.250	23.173	0.688	1.544	0.789	13.108
DeltaCt	Quadratic ridge	19.848	22.921	0.695	1.105	0.820	10.212
DeltaCt	Ridge	20.676	24.714	0.646	1.532	0.778	12.553
DeltaCt	Linear	21.247	25.568	0.621	1.706	0.824	10.381
DeltaDeltaCt	Quadratic ridge	21.589	26.415	0.596	2.081	0.806	11.644

Supplementary Material SM7: Global predictive performance of the tested models. Summary table of predictive performance across alternative data representations (raw Ct, ΔCt, ΔΔCt) and model classes, using leave-one-heart-out cross-validation.

SM8

Representation	Model	Genes	k	MAE_h	RMSE_h	R2	Bias_h	Calibration_slope	Calibration_intercept
Raw Ct	Linear	casp3+hmox1+hprt1	3	16.098	21.083	0.742	1.338	0.790	12.517
Raw Ct	Ridge	casp3+hif1a+hmox1+hprt1	4	16.187	20.332	0.760	0.808	0.761	13.500
Raw Ct	Ridge	casp3+hmox1+hprt1	3	16.210	20.905	0.746	1.095	0.738	14.997
Raw Ct	Linear	hmox1+hprt1	2	16.405	20.922	0.746	1.348	0.774	13.806
Raw Ct	Quadratic ridge	bax+hmox1+hprt1	3	16.806	21.281	0.737	1.467	0.741	15.071
Raw Ct	Linear	gapdh+hmox1+hprt1	3	16.895	21.902	0.721	1.442	0.844	10.198
Raw Ct	Linear	hif1a+hmox1+hprt1	3	16.915	21.519	0.731	1.772	0.810	10.889
Raw Ct	Quadratic ridge	casp3+hif1a+hmox1+hprt1	4	16.916	20.835	0.748	0.714	0.781	13.814
Raw Ct	Quadratic ridge	casp3+hmox1+hprt1	3	16.976	21.202	0.739	1.144	0.782	13.024
Raw Ct	Ridge	gapdh+hmox1+hprt1	3	17.074	21.364	0.735	0.622	0.771	13.651
Raw Ct	Ridge	hmox1+hprt1	2	17.103	21.730	0.726	1.212	0.721	16.410
Raw Ct	Ridge	hif1a+hmox1+hprt1	3	17.222	21.755	0.725	1.151	0.726	15.667
DeltaCt	Linear	casp3+gapdh+hmox1+hprt1	4	20.378	25.452	0.624	1.377	0.767	13.957
DeltaCt	Linear	gapdh+hif1a+hmox1+hprt1	4	20.425	25.444	0.624	1.621	0.768	12.924
DeltaCt	Ridge	casp3+gapdh+hmox1+hprt1	4	20.494	24.503	0.652	1.514	0.749	14.258
DeltaCt	Linear	casp3+hif1a+hmox1+hprt1	4	20.663	25.267	0.630	1.806	0.808	10.827

Representation	Model	Genes	k	MAE_h	RMSE_h	R2	Bias_h	Calibration_slope	Calibration_intercept
DeltaCt	Quadratic ridge	casp3+gapdh+hmox1+hprt1	4	20.720	24.695	0.647	1.454	0.802	10.482
DeltaCt	Ridge	gapdh+hif1a+hmox1+hprt1	4	20.873	24.715	0.646	1.539	0.730	14.914
DeltaCt	Ridge	casp3+hif1a+hmox1+hprt1	4	21.140	25.125	0.634	1.710	0.764	12.943
DeltaCt	Linear	gapdh+hmox1+hprt1	3	21.157	25.872	0.612	0.844	0.761	14.616
DeltaCt	Quadratic ridge	gapdh+hif1a+hmox1+hprt1	4	21.172	25.046	0.636	1.291	0.836	8.561
DeltaCt	Quadratic ridge	casp3+hif1a+hmox1+hprt1	4	21.346	25.336	0.628	1.285	0.793	11.324
DeltaDeltaCt	Linear	gapdh+hif1a+hmox1+hprt1	4	18.654	22.494	0.706	1.755	0.801	11.275
DeltaDeltaCt	Linear	gapdh+hmox1+hprt1	3	18.766	22.920	0.695	1.861	0.818	10.673
DeltaDeltaCt	Linear	hmox1+hprt1	2	18.857	22.667	0.701	1.888	0.808	11.643
DeltaDeltaCt	Linear	hif1a+hmox1+hprt1	3	18.905	22.873	0.696	1.539	0.836	10.520
DeltaDeltaCt	Quadratic ridge	hmox1+hprt1	2	19.067	23.108	0.689	1.948	0.737	15.903
DeltaDeltaCt	Quadratic ridge	hif1a+hmox1+hprt1	3	19.243	23.602	0.676	2.215	0.720	16.931
DeltaDeltaCt	Quadratic ridge	gapdh+hif1a+hmox1+hprt1	4	19.444	23.906	0.668	2.280	0.705	17.729
DeltaDeltaCt	Quadratic ridge	gapdh+hmox1+hprt1	3	19.593	23.743	0.672	2.241	0.715	17.002
DeltaDeltaCt	Ridge	gapdh+hif1a+hmox1+hprt1	4	19.617	23.528	0.678	1.687	0.727	16.835
DeltaDeltaCt	Ridge	hmox1+hprt1	2	19.685	23.470	0.679	1.827	0.716	17.062
DeltaDelt	Ridge	hif1a+hm	3	19.721	23.754	0.672	1.791	0.714	17.307

Representation	Model	Genes	k	MAE_h	RMSE_h	R2	Bias_h	Calibration_slope	Calibration_intercept
aCt		ox1+hprt1							
DeltaDeltaCt	Ridge	gapdh+hmox1+hprt1	3	19.951	24.297	0.657	1.749	0.719	17.592
Raw Ct	Linear	all 6 targets	6	18.477	23.961	0.667	1.634	0.808	11.849
DeltaCt	Linear	casp3+gapdh+hmox1+hprt1	4	20.378	25.452	0.624	1.377	0.767	13.957

Supplementary Material SM8: Performance of reduced gene panels. Comparison of predictive performance obtained with reduced multigene combinations, highlighting parsimonious panels capable of maintaining competitive accuracy for PMI estimation.

SM9

HeartID	PMI_h	Raw Ct: HMOX1 + HPRT1 Linear model	Raw Ct: all 6 targets Linear model	DeltaCt: CASP3 + GAPDH + HMOX1 + HPRT1 Linear model	Raw Ct: HMOX1 + HPRT1 Linear model / residual_h	Raw Ct: HMOX1 + HPRT1 Linear model / abs_error_h	Raw Ct: all 6 targets Linear model / residual_h	Raw Ct: all 6 targets Linear model / abs_error_h	DeltaCt: CASP3 + GAPDH + HMOX1 + HPRT1 Linear model / residual_h	DeltaCt: CASP3 + GAPDH + HMOX1 + HPRT1 Linear model / abs_error_h
H1	0	-14.774	-23.272	-17.289	-14.774	14.774	-23.272	23.272	-17.289	17.289
H1	12	14.998	-1.458	7.783	2.998	2.998	-13.458	13.458	-4.217	4.217
H1	24	22.555	13.813	26.419	-1.445	1.445	-10.187	10.187	2.419	2.419
H1	48	37.385	40.307	32.854	-10.615	10.615	-7.693	7.693	-15.146	15.146
H1	72	65.081	60.043	66.895	-6.919	6.919	-11.957	11.957	-5.105	5.105
H1	96	86.123	80.674	77.297	-9.877	9.877	-15.326	15.326	-18.703	18.703
H1	120	100.516	110.515	102.725	-19.484	19.484	-9.485	9.485	-17.275	17.275
H2	0	-11.300	-11.374	-18.468	-11.300	11.300	-11.374	11.374	-18.468	18.468
H2	12	9.860	7.618	0.084	-2.140	2.140	-4.382	4.382	-11.916	11.916
H2	24	20.975	14.576	20.239	-3.025	3.025	-9.424	9.424	-3.761	3.761
H2	48	33.497	29.914	31.772	-14.503	14.503	-18.086	18.086	-16.228	16.228
H2	72	71.819	65.988	65.700	-0.181	0.181	-6.012	6.012	-6.300	6.300
H2	96	90.187	81.212	76.837	-5.813	5.813	-14.788	14.788	-19.163	19.163
H2	120	96.428	100.792	92.499	-23.572	23.572	-19.208	19.208	-27.501	27.501
H3	0	-13.768	-19.900	-17.143	-13.768	13.768	-19.900	19.900	-17.143	17.143
H3	12	-8.819	-7.134	9.061	-20.819	20.819	-19.134	19.134	-2.939	2.939
H3	24	14.389	16.877	22.327	-9.611	9.611	-7.123	7.123	-1.673	1.673
H3	48	44.737	37.677	36.393	-3.263	3.263	-10.323	10.323	-11.607	11.607
H3	72	78.380	69.587	70.255	6.380	6.380	-2.413	2.413	-1.745	1.745
H3	96	84.839	78.856	76.983	-11.161	11.161	-17.144	17.144	-19.017	19.017
H3	120	90.910	98.892	90.681	-29.090	29.090	-21.108	21.108	-29.319	29.319
H4	0	-20.814	-18.559	-24.933	-20.814	20.814	-18.559	18.559	-24.933	24.933
H4	12	0.718	4.473	7.234	-11.282	11.282	-7.527	7.527	-4.766	4.766
H4	24	16.732	18.820	23.659	-7.268	7.268	-5.180	5.180	-0.341	0.341
H4	48	46.649	41.344	39.759	-1.351	1.351	-6.656	6.656	-8.241	8.241
H4	72	63.106	58.604	61.804	-8.894	8.894	-13.396	13.396	-10.196	10.196
H4	96	72.685	68.928	65.369	-23.315	23.315	-27.072	27.072	-30.631	30.631
H4	120	99.290	95.087	89.761	-20.710	20.710	-24.913	24.913	-30.239	30.239
H5	0	-14.281	-9.345	-15.673	-14.281	14.281	-9.345	9.345	-15.673	15.673
H5	12	3.448	-0.288	4.948	-8.552	8.552	-12.288	12.288	-7.052	7.052
H5	24	23.524	18.663	18.050	-0.476	0.476	-5.337	5.337	-5.950	5.950
H5	48	51.795	39.909	42.510	3.795	3.795	-8.091	8.091	-5.490	5.490

HeartID	PMI_h	Raw Ct: HMOX1 + HPRT1 Linear model	Raw Ct: all 6 targets Linear model	DeltaCt: CASP3 + GAPDH + HMOX1 + HPRT1 Linear model	Raw Ct: HMOX1 + HPRT1 Linear model / residual_h	Raw Ct: HMOX1 + HPRT1 Linear model / abs_error_h	Raw Ct: all 6 targets Linear model / residual_h	Raw Ct: all 6 targets Linear model / abs_error_h	DeltaCt: CASP3 + GAPDH + HMOX1 + HPRT1 Linear model / residual_h	DeltaCt: CASP3 + GAPDH + HMOX1 + HPRT1 Linear model / abs_error_h
H5	72	66.450	58.414	61.137	-5.550	5.550	-13.586	13.586	-10.863	10.863
H5	96	82.064	76.430	72.267	-13.936	13.936	-19.570	19.570	-23.733	23.733
H5	120	103.323	108.101	90.984	-16.677	16.677	-11.899	11.899	-29.016	29.016
H6	0	-14.607	-17.018	-16.691	-14.607	14.607	-17.018	17.018	-16.691	16.691
H6	12	1.130	-0.138	7.265	-10.870	10.870	-12.138	12.138	-4.735	4.735
H6	24	15.837	15.953	20.637	-8.163	8.163	-8.047	8.047	-3.363	3.363
H6	48	49.287	41.688	40.054	1.287	1.287	-6.312	6.312	-7.946	7.946
H6	72	71.814	58.527	61.657	-0.186	0.186	-13.473	13.473	-10.343	10.343
H6	96	81.851	73.798	69.011	-14.149	14.149	-22.202	22.202	-26.989	26.989
H6	120	95.798	101.562	92.649	-24.202	24.202	-18.438	18.438	-27.351	27.351
H7	0	-15.344	-18.039	-15.626	-15.344	15.344	-18.039	18.039	-15.626	15.626
H7	12	7.507	5.610	10.867	-4.493	4.493	-6.390	6.390	-1.133	1.133
H7	24	20.822	17.427	21.777	-3.178	3.178	-6.573	6.573	-2.223	2.223
H7	48	39.426	33.865	40.752	-8.574	8.574	-14.135	14.135	-7.248	7.248
H7	72	69.080	63.951	67.043	-2.920	2.920	-8.049	8.049	-4.957	4.957
H7	96	84.396	77.222	72.791	-11.604	11.604	-18.778	18.778	-23.209	23.209
H7	120	92.750	96.863	89.465	-27.250	27.250	-23.137	23.137	-30.535	30.535
H8	0	-11.580	-8.523	-17.970	-11.580	11.580	-8.523	8.523	-17.970	17.970
H8	12	8.234	4.276	4.531	-3.766	3.766	-7.724	7.724	-7.469	7.469
H8	24	15.683	13.568	20.848	-8.317	8.317	-10.432	10.432	-3.152	3.152
H8	48	42.072	34.718	38.100	-5.928	5.928	-13.282	13.282	-9.900	9.900
H8	72	68.341	61.859	67.265	-3.659	3.659	-10.141	10.141	-4.735	4.735
H8	96	82.643	75.555	70.830	-13.357	13.357	-20.445	20.445	-25.170	25.170
H8	120	98.812	99.578	90.151	-21.188	21.188	-20.422	20.422	-29.849	29.849
H9	0	-12.478	-14.834	-14.993	-12.478	12.478	-14.834	14.834	-14.993	14.993
H9	12	12.463	7.748	8.596	0.463	0.463	-4.252	4.252	-3.404	3.404
H9	24	20.029	17.181	22.117	-3.971	3.971	-6.819	6.819	-1.883	1.883
H9	48	37.667	35.088	38.005	-10.333	10.333	-12.912	12.912	-9.995	9.995
H9	72	69.235	62.579	67.794	-2.765	2.765	-9.421	9.421	-4.206	4.206
H9	96	85.935	77.933	73.554	-10.065	10.065	-18.067	18.067	-22.446	22.446
H9	120	96.489	101.533	92.599	-23.511	23.511	-18.467	18.467	-27.401	27.401

Supplementary Material SM9: Individual predictions and residuals of the selected models. Observed and predicted PMI values for the selected models, with corresponding residuals and absolute errors for each sample in leave-one-heart-out validation.
